# Images dataset of beef meat samples with different shelf life

**DOI:** 10.1016/j.dib.2023.109503

**Published:** 2023-08-20

**Authors:** Julieta Domínguez-Soberanes, María T. Orvañanos-Guerrero, Claudia N. Sánchez, Maximiliano Lara, Esteban García, Juan Pablo Cisneros, Luis Enrique Orozco, Ernesto Rosales-Tavera

**Affiliations:** a Universidad Panamericana, Facultad de Ingeniería, Aguascalientes, 20296, México; b Universidad Panamericana, Escuela de Dirección de Negocios Alimentarios, Aguascalientes, 20296, México

**Keywords:** Beef meat images, Beef meat color, Inside skirt images, Knuckle images, Sirloin images

## Abstract

Three different cuts of meat samples: inside skirt, knuckles, and sirloin were picture captioned on the first and fifth day after purchase. From each type of meat cut, ten pictures were taken at the beginning and the end of the studied shelf life, obtaining 60 different images. The images were taken under control variables in a black acrylic cabin. In addition to the original images, we proportionate another set of 60 processed images. The latter were obtained after color calibration and meat segmentation. All these images could be used for future experiments where the color in meat should be analyzed.

Specifications Table

**Table:** 

Subject	Food Science
Specific subject area	Analysis of meat quality through color analysis
Type of data	Images in jpg format
How the data were acquired	Images were captured using the digital camera Sony Cyber-Shot W830 inside a cabin. The cabin is 3 mm thick, black acrylic, and has two LED light tubes that keep the internal light of the booth constant at 640 lm. In addition, it has a mechanism that allows the position of the camera and meat samples to be kept constant, granting freedom of movement in the Z-axis to move the samples away from and closer to the camera. The movement in Z is achieved with a shelving system in which a base can slide every 5 mm. The cabin size was decided based on the measurement of the meat containers. It has a particular part on the top that keeps the camera in the same place. All cabin parts are washable and waterproof to allow proper hygiene.
Data format	Raw Analyzed
Description of data collection	Thirty pieces of beef meat were captured in the images. Three cuts were considered: inside skirt, knuckles, and sirloin. We used ten pieces for each cut (5 cm by 5 cm with 0.5 cm thickness). Images were captured on the first and the fifth day after purchase. In addition to the original images, we proportionate the set of processed images. The latter were obtained after color calibration and meat segmentation. In total, data consists of 120 images (1030 x 772 pixels), 60 of them are the originally captured images, and the remaining 60 correspond to the processed ones. Half of the images correspond to fresh meat after purchase, and the other half correspond to non-fresh meat after five days of purchase.
Data source location	Institution: Universidad Panamericana City/Town/Region: Aguascalientes/Aguascalientes/Bajío Country: México
Data accessibility	Repository name: Images of fresh and non-fresh beef meat samples DOI data: 10.17632/wvhkpppddp.1 Direct URL to data: https://data.mendeley.com/datasets/wvhkpppddp/1
Related research article	Claudia N. Sánchez, María Teresa Orvañanos-Guerrero, Julieta Domínguez-Soberanes, Yenizey M. Álvarez-Cisneros. Analysis of beef quality according to color changes using computer vision and white-box machine learning techniques. Heliyon (2023), Volume 9, Issue 7. doi.org/10.1016/j.heliyon.2023.e17976.

## 1 Value of the Data

• The dataset presents meticulously captured images of beef meat samples, showcasing two stages of shelf life for three different cuts, so the images can serve as a color reference for identifying beef meat quality.
• Scientists and food technologists can exploit this dataset to advance quality assessment techniques. The controlled imaging environment ensures consistent and reliable data, allowing researchers to create more precise algorithms, models, and color analysis tools, thus improving assessment efficiency.
• Meat companies and retailers can use this dataset to establish enhanced guidelines for evaluating meat freshness before distribution, cutting waste, and elevating customer satisfaction.
• In academics, it can show color analysis's importance in food quality assessment, demonstrating practical data collection, processing, and real-world application.

## 2 Data Description

**Fig. 1:**
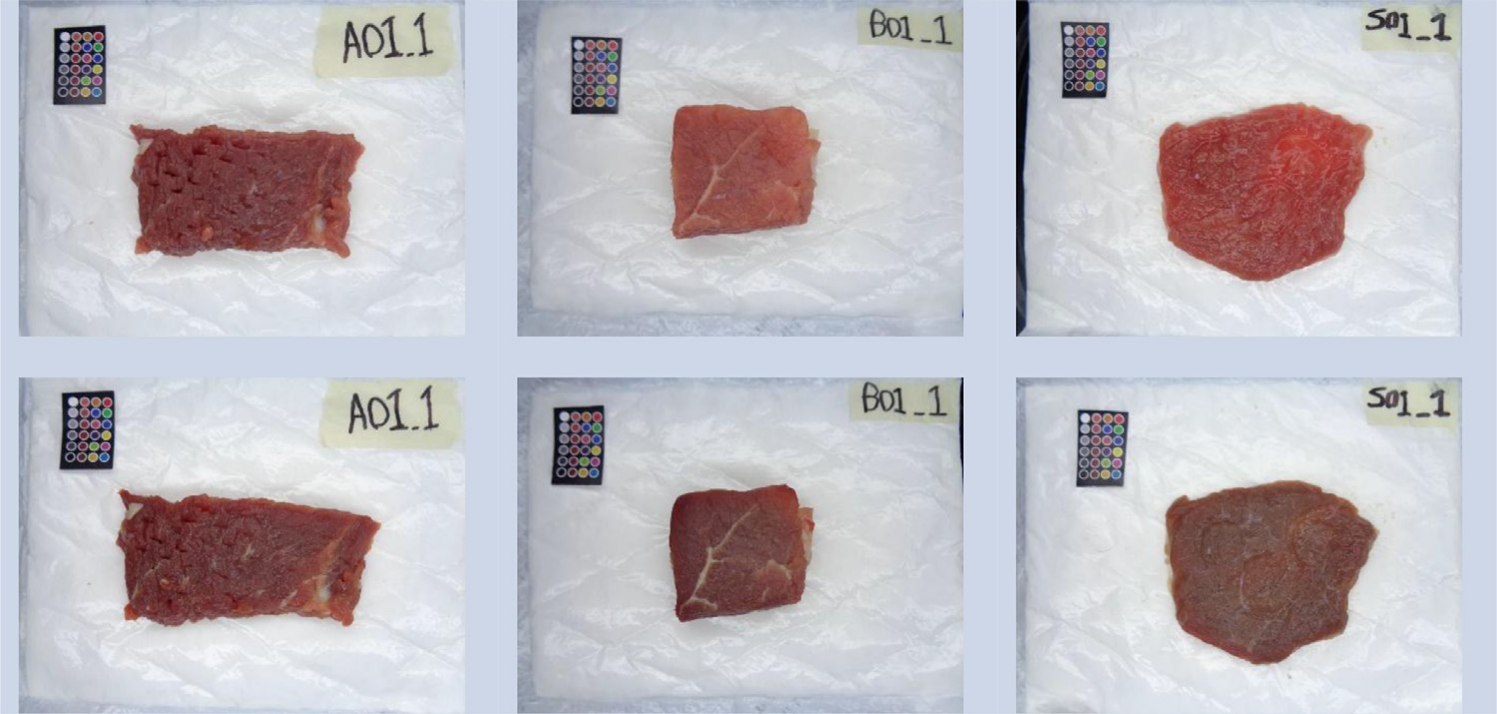
shows examples of the originally captured images. They include the sample code (up-right) and the color checker matrix that can be used for color calibration. The sample letter in the sample code corresponds to the cut: inside skirt (A), knuckles (B), and sirloin (S). The first row corresponds to the first day and the second one to the fifth day after purchase.

**Fig. 2:**
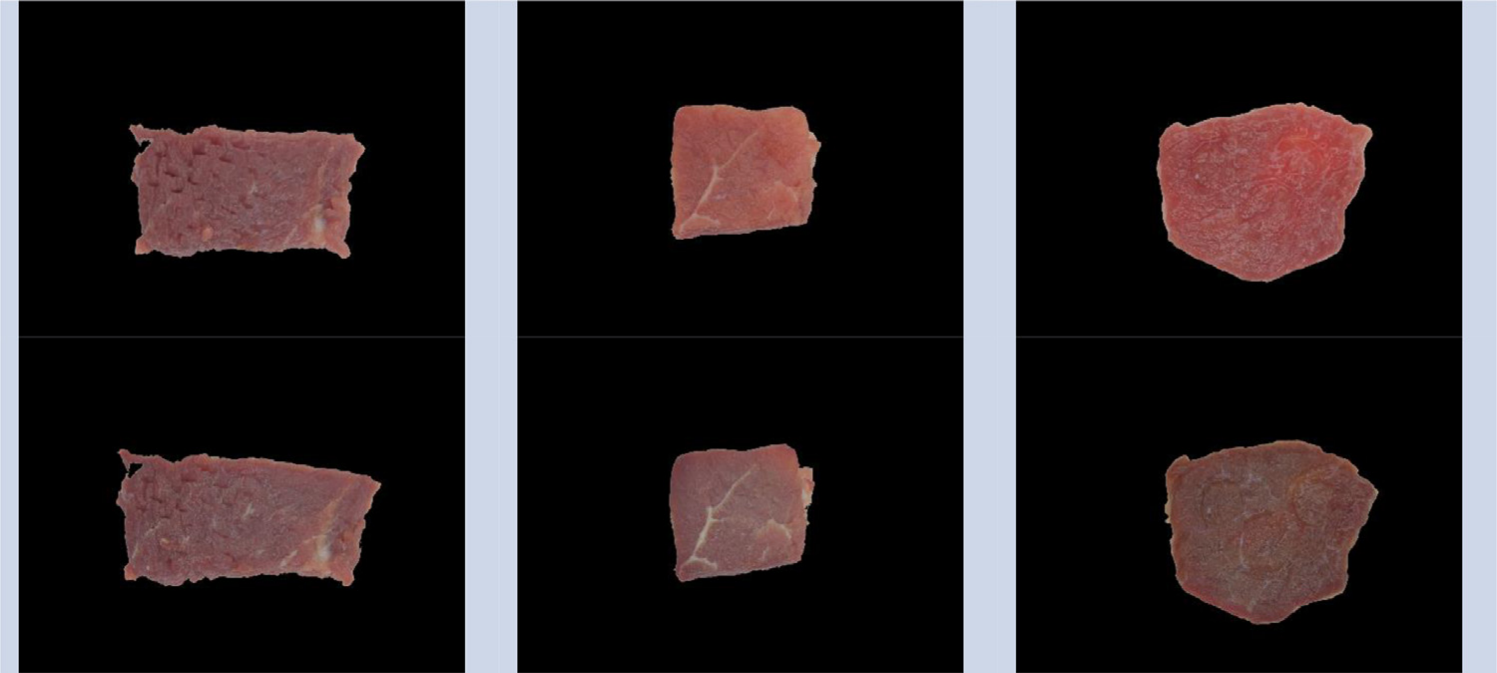
Beef meat samples after color calibration and meat segmentation. The first row corresponds to the first day and the second one to the fifth day after purchase. Columns, from left to right, represent samples of inside skirt, knuckle, and sirloin, respectively.

This dataset presents several images for analyzing the color of beef meat samples with different shelf life. Thirty pieces of beef meat were captured. Three cuts were considered: inside skirt, knuckles, and sirloin. We used ten pieces for each cut (5 cm by 5 cm). Images were captured on the first and the fifth day after purchase. In total, the dataset consists of 120 images (1030 x 772 pixels), 60 of them are the originally captured images (see Fig. 1), and the remaining 60 correspond to the processed ones (see Fig. 2). Half of the images correspond to fresh meat after purchase, and the other half correspond to non-fresh meat after five days of purchase.

In addition to the original images, we proportionate the set of processed images. They were obtained after color calibration and meat segmentation. Fig. 2 shows an example of these images. In fact, the meat samples correspond to the same presented in Fig. 1.

All the data is freely available on Mendeley Data in the repository Images of fresh and non-fresh beef meat samples (https://data.mendeley.com/datasets/wvhkpppddp/1). On this repository, the files are called with the following structure. YYYYMMDD_[Cut]_[Sample].jpg. YYYY represents the year, MM the month, and DD the day. We have only three different cuts: inside skirt (A01), knuckle (B01), and sirloin (S01). The sample corresponds to the number of samples for a specific cut, varying from 1 to 10. For example, 20210830_A01_10.jpg corresponds to an image of the tenth sample of inside skirt, and the picture was taken the August 30th, 2021. In addition, we have some images that correspond to the segmented versions, these are marked with _seg at the last of the file names. The samples of inside skirt corresponding to the first and fifth day are the ones that start with 20210830 and 20210903. The samples of sirloin corresponding to the first and fifth day are the ones that start with 20210906 and 20210910. Finally, the samples of knuckle corresponding to the first and fifth day are the ones that start with 20210913 and 20210917.

## 3 Experimental Design, Materials and Methods

### 3.1 Meat sample selection

Three meat samples were used in the experiment: a) inside skirt, b) knuckles, and c) sirloin. We chose the meat cuts based on the hypothesis that the color of the meat muscle is based on different parameters such as cattle`s genetics, feed, age, gender, and part of the cattle from which the cut was obtained [1]. Moreover, this dataset focuses on changes in the color of the meat during its shelf life because myoglobin is oxygenated due to exposure to air, and therefore, this protein is converted into metmyoglobin [2]. In this case, we chose a shelf life of 5 days.

The meat was purchased at a local store in Aguascalientes (Tepeyac), Mexico, during the summer of 2021 ( August-September). The inside skirt was purchased on August 4th, the sirloin on September 6th, and the knuckle on September 13th. The supplier transported the meat to Universidad Panamericana, where an expert received it, weighed it, and refrigerated it at 4 °C. Then it was cut, using a stainless steel knife, with the meat pieces sliced by students soon to become chefs, therefore considered experts. They asked to cut the meat into dimensions of approximately 5 cm in length by 5 cm in width and thickness of  0.5 cm. Subsequently, the pieces were stored in trays for storage. The samples were maintained at 4 °C throughout the studied shelf life and were covered with Petrifilm to reduce deterioration due to oxidation.

### 3.2 Image caption

All the images were taken around the same hour (2 pm) in a cabin that our research group designed in order to use the same light intensity in all the pictures that were captured. The cabin was 3 mm thick and designed with black acrylic. In the inner part of the cabin, there was a light of 640 lm. The camera, Sony Cyber-Shot W830 with a high-resolution of 20.1MP, was adjusted to the mechanism that allowed freedom of movement. It has a 1/2.3-inch sensor with dimensions of 6.17 × 4.55 mm and sensor area of 28.07mm2.

For these images, the samples were cut into 5 cm × 5 cm and were placed in a tray that could hold this type of size samples. Then they were put into the cabin with a color-checker used for calibration, which will be explained in the following section.

### 3.3 Color correction

The analysis of meat color is challenging because many optical factors can affect it. For example, objects' colors may be perceived differently if the illumination sources differ or several devices are used. The first factor could be affected by variables such as the hour or the place where the pictures were taken. On the second factor, different devices can vary objects' intensities or hues. In order to avoid those problems, we captured all the images in a cabin. Besides, we incorporated into all the images a color checker matrix (see Fig. 1) to perform a color correction of images. The original color checker matrix has a column useful for calibrating natural colors, such as human skin, vegetation, or the sky. In this case, we decided to replace that column with the second column containing meat color samples. The colors in the color checker matrix in hexadecimal format are #FFFFFF, #D2715D, #D67E2C, #FF0000, #C8C8C8, #BB5F4F, #505BA6, #00FF00, #A0A0A0, #A3463B, #C15A63, #0000FF, #7A7A7A, #954238, #5E3C6C, #E7C71F, #555555, #8B3D3C, #9DBC40, #BB5695, #000000, #793B2B, #E0A32E, #0885A1.

The first step for correcting the color is getting the 24 color values corresponding to the color checker matrix in the images. In this dataset, we decided to use the median of colors (RGB space) of the pixels in the circles. In this sense, we obtained a matrix $M_{RGB}\in R^{24x3}$  that contains 24 rows and 3 columns, where each row represents a color in the color checker matrix, and the columns represent the R, G, and B channels.

In general, to perform the color correction, we need a matrix that we called $M{{}^{\prime }}_{RGB}\in R^{24x3}$ that represents the colors that correspond to the calibrated ones. In addition to the matrix representing the colors in the color checker matrix of images MRGB. With both matrices, we can model a function F that can transform and calibrate the colors in the image (see Eq. (1)).

(1) $$M{{}^{\prime }}_{RGB}=\;F\left(M_{RGB}\right)$$

We used the library color [3] to implement the color correction. Two techniques were tested, Cheung [4] and Vandermonde [5].

For measuring the errors, we had an image of the color checker matrix captured in the cabin. Then, we used the Mean Absolute Error among the colors of that image and the images used in the experiments. Table 1 presents the errors in colors before and after calibration. It can be seen that color correction significantly reduces the error among the colors in the images, being Vandermonde the technique with the best results. Knowing the range of pixels colors is between 0 and 255, the errors are small enough. In addition, using the ANOVA test, we found that the difference among the errors before and after correction (using Vandermonde) is statistically significant in all the cases, with a p-value of 9.33e-66, 3.34e-40, and 3.29e-62 for inside skirt, knuckle, and sirloin, respectively.

**Table 1:** Mean Absolute Error of colors before (original images) and after color calibration (Cheung and Vandermonde). The values are calculated based on the RGB color space in a range between 0 and 255.

	Inside Skirt	Knuckle	Sirloin
Original images	8.72	11.49	11.79
Cheung	6.49	5.02	7.93
Vandermonde	4.37	2.90	5.05

### 3.4 Image segmentation

**Fig. 3:**
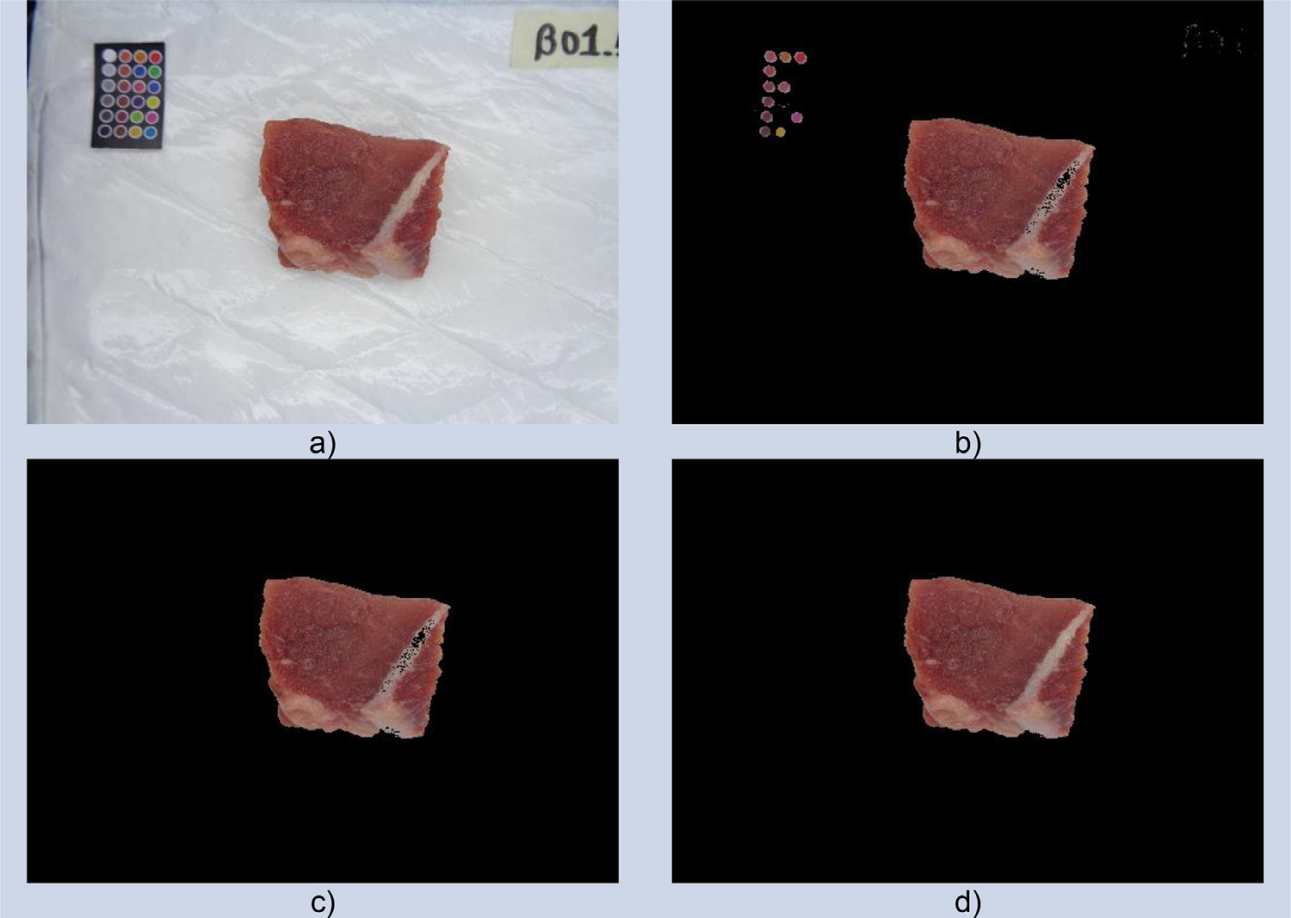
Example of the image segmentation. a) Original image. b) Image after pixel classification with SVM. c) Bigger connected component. d) Final image after binary fill holes. Image taken from [8].

Image segmentation consists of dividing an image into regions with similar features [6]. In this case, the goal is to keep only the meat region. The image segmentation process is described as follows (see Fig. 3). First, we implemented a binary classifier using a Support Vector Machine (SVM) [7]. On the one hand, we had the pixels that belonged to meat, and on the other hand, the pixels that belonged to the color checker matrix and the absorbent paper towels. This model allows us to identify the pixels with colors similar to meat (see Fig. 3.b). Then, we calculated the connected components, where in all cases, the meat corresponded to the bigger one (see Fig. 3.c). Finally, we used binary fill holes to obtain the whole meat area (see Fig. 3.d).

## 4 Conclusions and Future Studies

Nowadays, the meat industry, academics, and food scientists expect to offer the best quality of their products that their customers will consume. Therefore, this dataset collects different stages of shelflife, considering distinct cuts, serving as a future guideline for discerning meat beef. Using this information can be the first step in establishing meat quality guidelines, translating into greater customer satisfaction.

This study can be used to create algorithms, mathematical models, and innovative color analysis tools. Moreover, this study can be used to elevate the quality of assessment procedures for the Food Industry. In future research, we would like to extend the number of samples using all the commonly used beef meat cuts. Furthermore, we will consider taking more pictures and/ or measurements within the shelf life.

## Ethics Statements

The work does not involve human subjects, animal experiments, or any data collected from social media platforms.

## CRediT authorship contribution statement

**Julieta Domínguez-Soberanes:** Conceptualization, Methodology, Writing – original draft, Funding acquisition, Investigation, Writing – original draft, Project administration. **María T. Orvañanos-Guerrero:** Conceptualization, Methodology, Investigation, Funding acquisition, Project administration. **Claudia N. Sánchez:** Conceptualization, Methodology, Project administration, Funding acquisition, Investigation, Software, Data curation, Writing – original draft. **Maximiliano Lara:** Software, Data curation, Investigation. **Esteban García:** Software, Data curation, Investigation. **Juan Pablo Cisneros:** Data curation, Investigation. **Luis Enrique Orozco:** Data curation, Investigation. **Ernesto Rosales-Tavera:** Data curation, Investigation.

## Declaration of Competing Interest

The authors declare that they have no known competing financial interests or personal relationships that could have appeared to influence the work reported in this paper. However, it is essential to mention that the cabin described in the methodology has started a registration process as a utility model.

## Data Availability

Images of fresh and non-fresh beef meat samples (Original data) (Mendeley Data).
